# Experimental and empirical investigation of a CI engine fuelled with blends of diesel and roselle biodiesel

**DOI:** 10.1038/s41598-021-98382-1

**Published:** 2021-09-22

**Authors:** Tikendra Nath Verma, Upendra Rajak, Abhishek Dasore, Asif Afzal, A. Muthu Manokar, Abdul Aabid, Muneer Baig

**Affiliations:** 1 Department of Mechanical Engineering, Maulana Azad National Institute of Technology Bhopal, Bhopal, 462003 India; 2 School of Mechanical Engineering, RGM College of Engineering and Technology, Nandyal, 518501 India; 3 Department of Mechanical Engineering, P. A. College of Engineering (Affiliated To Visvesvaraya Technological University, Belagavi), Mangaluru, 574153 India; 4 Department of Mechanical Engineering, B. S. Abdur Rahman Crescent Institute of Science and Technology, Vandalur, Chennai, Tamil Nadu 60 0 048 India; 5 Engineering Management Department, College of Engineering, Prince Sultan University, PO BOX 66833, Riyadh, 11586 Saudi Arabia; 6 Department of Mechanical Engineering, School of Technology, Glocal University, Delhi-Yamunotri Marg, SH-57, Mirzapur Pole, Saharanpur, Uttar Pradesh 247121 India

**Keywords:** Energy science and technology, Engineering, Chemical engineering, Mechanical engineering

## Abstract

The continuous rise in demand, combined with the depletion of the world's fossil fuel reserves, has forced the search for alternative fuels. The biodiesel produced from Roselle is one such indigenous biodiesel with tremendous promise, and its technical ability to operate with compression ignition engines is studied in this work. To characterize the fuel blends, researchers used experimental and empirical approaches while operating at engine loads of 25, 50, 75, and 100%, and with fuel injection timings of 19°, 21°, 23°, 25°, and 27° before top dead center. Results indicate that for 20% blend with the change of injection timing from 19° bTDC to 27° bTDC at full load, brake specific fuel consumption and exhaust gas temperature was increased by 15.84% and 4.60% respectively, while brake thermal efficiency decreases by 4.4%. Also, an 18.89% reduction in smoke, 5.26% increase in CO_2,_ and 12.94% increase in NOx were observed. In addition, an empirical model for full range characterization was created. With an r-squared value of 0.9980 ± 0.0011, the artificial neural network model constructed to characterize all 10 variables was able to predict satisfactorily. Furthermore, substantial correlation among specific variables suggested that empirically reduced models were realistic.

## Introduction

Alternative fuel research is necessitated by the limited supply and serious environmental challenges associated with the use of non-renewable energy sources. Since Rudolf Diesel invented biodiesel in 1902, using oils derived from locally accessible crops, global biodiesel consumption has increased to 13–14 percent of total fuel energy consumption^1^. Numerous researchers have indicated that the esters resulting from certain non-edible oils could aid as an alternate fuel^2^. Also, it has been revealed that lower biodiesel blends display very similar characteristics as that of standard diesel fuel. In general, as compared with diesel fuel, neat biodiesel fuel and its blends have higher density and kinematic viscosity, lower calorific value, and volatility^3,4^. In consequence, these different critical physicochemical properties could yield different engine characteristics. Differences in fundamental operational properties such as atomization of fuel, ignition delay, and rate of fuel mass-burn, etc. could directly result in different performance, combustion, and emission behavior.

The fuel injection conditions are a major influencing factor in the operation of an internal combustion engine (ICE). A pre-metered amount of charge is to be injected at the end of the compression stroke during the cyclic operation^5^. The timing at which the fuel is injected into the combustion chamber is referred to as the fuel injection timing (FIT) and is measured as crank angle before top dead center (bTDC). And, the associated pressure at which the charge is injected is referred to as fuel injection pressure (FIP). Advanced injection of charge would facilitate more burning of charge before the piston reaches TDC and consequently, maximum cylinder pressure would also be observed at advanced FIT. Such advanced FIT may result in inefficiency of the engine as sufficient temperature may not be available at the beginning of combustion^6,7^. It has also been noticed that retardation in fuel injection timing could reduce NOx emission without any significant impact on the engine performance^8,9^. Given the fundamental role of fuel injection conditions in the behavior of this heat engine, the following section discusses what recent researches have reported on how FIT influences ICE performance, combustion, and emission characteristics^10^.

### Effects of FIT

The process of controlled combustion of fuel inside the combustion chamber is governed by thermodynamic variables such as temperature and pressure. Even at extremely tiny length and time scales, such variables have a significant impact on the combustion process. Following the fuel injection, the velocity must be high enough to allow for proper atomization and mass dispersion throughout the combustion chamber. As a result, the timing of fuel injection, in addition to the pressure at which fuel is injected, controls the evolution of the combustion process. As the piston head approaches TDC for the ensuing power stroke, advancement or retardation of the fuel injection timing impacts the evolution of combustion, in addition to a wide number of other variables.

To report a few recent investigations on the effects of only FIT on engine behavior, Suryawanshi and Deshpande^11^ studied the effect of decreased FIT by 4° CA on the performance and emission of a Karanja oil methyl ester fueled in CI engine. The addition of biodiesel in the fuel blend caused a significant reduction in smoke, hydrocarbon, and carbon monoxide emission but there was a small increase in NOx emission with a standard FIT. Similarly, Nwafor^12^ studied the effect of increasing FIT by 3.5° CA bTDC on the engine exhaust fueled with natural gas as a primary fuel in dual-fuel CI engine emission and performance. The experiment test engine had original FIT of 30° CA bTDC. The results showed that increased FIT reduced BTE, CO_2,_ and CO. Numerous authors also reported that retarding the FIT reduced NOx emission^13–15^. On the contrary, Rahman et al.^16^ reported an increase in HC, CO, and smoke emission increases while NOx emission decreases with retardation in FIT. These types of fuel and operating condition-specific observations were also reported by many authors where BSFC and EGT increased while BTE decreased with advanced FIT^17^.

To extend these engine characterization with operating conditions defined by more than FIT, the following investigations studied the combined effect of fuel injection conditions with CR. Along with an increase/decrease in FIT, Raheman and Ghadge^18^ observed that BSFC decreases while BTE ad EGT increases with an increase in CR from 18 to 20. Laguitton et al.^19,20^ also examined the effects of FIT and CR for different biodiesels. The study reported that there was no major deviation in the performance and combustion behavior, but a small reduction in NOx and CO_2_ emission and increase in smoke emission were observed with retarding FIT and decreasing CR. Such observations were also reported by Sayin and Gumus^21^ where the effect of CR, FIT, and FIP on emission and performance with biodiesels were studied. A comprehensive observation from the study was that engine performance and NOx increased with increase in CR, FIP, and FIT. Such observations of the combined effect of CR and fuel injection conditions were also reported in few other studies^22^.

On extended studies involving optimization of engine operation, some authors have reported that the optimal FIT for different biodiesel depended on engine inputs of engine torque, engine speed, and fuel injection duration^23–25^ thus consolidating the role of fuel injection conditions in the ICE operation. Possibly, advancing in FIT could greatly influence the combustion duration and therefore could change combustion duration which could lead to either successful combustion or incomplete combustion^26,27^. On another account involving ignition delay (ID) along with fuel injection conditions, Bari et al.^28^ reported that ID was higher with advanced FIT. It was speculated that proper mixing of the air–fuel mixture was promoted inside the combustion chamber with advanced FIT, and thus facilitating better fuel combustion in the premixed combustion zone.

As demonstrated by these few reported studies, what is clearly evidenced is that the characterization of engine behavior posits as a challenge when all the operating conditions are to be accounted for. The thermochemical interactions entirely change when the physicochemical properties of the fuel changes. Therefore, it is a necessity to investigate how different fuels with different properties behave under specific operating conditions. In this regard, we investigated how the indigenous biodiesel from Roselle oil behaved under operating conditions largely defined by critical parameters of fuel injection conditions.

### Artificial neural network

The activation or inhibition of extensive networks of neurons is generally credited to how humans are able to learn many movements in the learning paradigm in human movement studies. Such networks have a one-of-a-kind ability to learn and grow features from prior moves and apply them to new situations. To study how these networks of neurons are able to conform to patterns of the inputs^29^ networks of artificial neurons were designed. Popularly known as artificial neural networks (ANNs), they have find substantial application for classification and prediction across numerous domains^30^ in artificial intelligence and machine learning. As a supervised machine learning algorithm, it maps the input to the target values through iterative weights and biases adjustment and hence usually performs satisfactorily when large enough dataset is available. During the training, the adjustment of coefficient allows the network to develop plausible empirical patterns of the dataset. But such patterns cannot explain the mechanistic relationship between the involved parameters, and hence they are treated as ‘black-box’ models. As such the drawback might be, in the research of alternative fuels operating with internal combustion engines (ICE), the lack of unified analytical relationships has only substantiated why alternative approaches to modelling ICE are required. It is evidenced by how the following previous researches have employed ANN to achieve the prediction of several variables of interest involved in ICE research.

To report a few, Alonso et al.^31^ used ANN to develop models for the ICE operation with diesel for the optimization of performance and emission using genetic algorithm. The ANN model had inputs of engine operating speed, mass of air and injected fuel, fuel injection conditions, and temperature of water and intake. It predicted emission parameters of NOx, PM, CO, HC, and BSFC. A more computationally intensive study was performed for ICE operation with blends of WCO by Ghobadian et al.^32^, where various ANN were evaluated for best prediction accuracy. Using speed and blending, the network predicted brake power, BSFC, torque, HC, and CO emissions. Similar studies were also reported by Togun and Baysec^33^. They also used ANN to develop torque and BSFC from operating parameters of spark advance, throttle position, and engine speed. Similar study for blends with WCO biodiesel was conducted by Pai and Rao^34^ where load, blend, CR, and FIT were used to predict BTE, EGT, BSEC, smoke, NOx, and UHC. In addition to ANN model using brake power and blending to predict BTE, BSFC, CO, smoke, NOx, and HC, Sharon et al.^35^ presented a SIMULINK representation of the model.

Statistical flair to the paradigm was added by Roy et al.^36^ where many statistical parameters were incorporated to evaluate the network’s prediction accuracy. The study predicted BTE, BSFC, NOx, CO_2_, PM from load, EGR, fuel injection pressure, and injected fuel mass. In addition with a more empirical perspective^37^, coupled ANN model with limited solutions derived from detailed numerical solutions to highlight that large computational resources can be saved thus. Predicting 17 variables of performance, combustion, and emission, the study also reported empirical redundancy thus indicating plausible reduced empirical models of ICE operation. As evidenced from these few studies, ANN has been popularly employed to build empirical models of ICE operation. It has aided in the prediction of approximated engine behavior, which is required in a number of industrial applications such as fast diagnostics and defect identification. However, it is also clear that these studies did not investigate the system's empirical structure. As a result, we proposed in this work to assess the dataset's empirical redundancy in order to analyze viable empirically reduced models of the system.

As a result, the technical viability of Roselle biodiesel and its binary mixes with diesel as a working fuel in a DI CI engine is evaluated in the current study. Using the transesterification process, biodiesel was produced from Roselle seeds. LA20, LA40, LA60, LA80, and LA100 sample blends, as well as pure diesel fuel, are available for testing. These samples were tested in a conventional bench scale CI engine at 19°, 21°, 23°, 25°, and 27° bTDC FITs and at 25 percent, 50 percent, 75 percent, and 100 percent engine loading. The results of the studies are used to investigate the engine's overall characteristics. Finally, this experimental data is used to create an ANN-based empirical model. Analyzing the analysis of these empirical engine reactions, realistic empirically reduced models were investigated.

## Experimental procedure

### Fuel preparation

Roselle oil is an edible oil attained from Roselle (*Hibiscus sabdariffa L.*) seeds and 96.5% of oil can be extracted from roselle seeds^41^. One litre of crude Roselle oil was taken in a repository and heated to a temperature around 65–70 °C at low stirring speed. In a separate flask, a fixed quantity of alcohol (methanol) and sodium hydroxide (NaOH) as a catalyst was strenuously shaken and poured into a container and was closed with an air-tight lid. The sample was mixed for an hour at minimum speed by the use of a magnetic stirrer and then it was transferred into a separation funnel to allow to settle overnight at room temperature^38,39,52^. The Roselle oil is turned into biodiesel form and glycerol is separated through the funnel. The projected production cost of Roselle biodiesel is 0.92 $ per liter^41^. The biodiesel floated at the top and the glycerol stayed at the bottom. The separated crude biodiesel phase was then washed out with warm deionized water until the washed water becomes clear. The evaporation process then removed the remaining water under atmospheric conditions.

The requirements for the production of Roselle biodiesel using the transesterification process are shown in Table 1. The total biodiesel yield from Roselle oil produced with the NaOH catalyst is 83 percent. Using gas chromatography-mass spectrometry, the fatty acid content was measured under ideal conditions. The fatty acid composition of Roselle oil is shown in Table 2. In the analytical laboratory, the key parameters of fuel blend and diesel were determined according to ASTM standards. The important properties of different tested fuels are shown in Table 3.

**Table 1:** Transesterification reaction requirement for the production of Roselle biodiesel.

Description	Values
Oil quantity	1000 ml
Methanol (1:3 molar ratio)	280 ml
Sodium hydroxide (NaOH)	1.65 ml
Temperature	60–75 °C
Time period	60–70 min
RPM	320–600 rpm
Biodiesel obtained	830 ml

**Table 2:** Free fatty acid composition of Roselle biodiesel.

Fatty Acid	Structure	Molecular structure	Formula	Mol. wt	% (w/w)
Linoleic acid	18:2		C_18_H_32_O_2_	280.2512	38.18
Palmitic acid	16:0		C_16_H_32_O_2_	257.2503	18.49
Linolenic acid	18:3		C_18_H_30_O_2_	279.2235	2.08
Oleic acid	18:1		C_18_H_34_O_2_	281.2667	33.32
Lignoceric acid	24:0		C_24_H_48_O_2_	367.3543	1.11
Stearic acid	18:0		C_18_H_36_O_2_	285.2624	4.08

**Table 3:** Important physical–chemical properties of diesel, Roselle and its blends.

Fuel	Testing method	B0	LA20	LA40	LA60	LA80	LA100
Density (@20°C) (kg/m^3^)	ASTM D4052	830	838.2	849.6	859.2	868.6	877
Kinematic viscosity (@40°C) (mm^2^/s)	ASTM D445	2.9	3.24	3.5	4.25	4.85	5.64
Heating value (MJ/ kg)	ASTM D4809	42.5	41.6	40.7	40.16	39.44	38.74
Cetane number	ASTM D613	48	48.8	49.67	50.45	51.25	52.3
Flash point (°C)	ASTM D93	50	75.2	94.4	116.7	135.2	159.2
C%	ASTM D5291	86.14	84	83.09	81.52	80.10	78.71
H%	ASTM D5291	13.86	13.49	13.12	12.75	12.29	12.12
O%	ASTM D5291	0.004	2.25	4.02	5.71	7.54	9.23

### Experimental procedure

**Figure 1:**
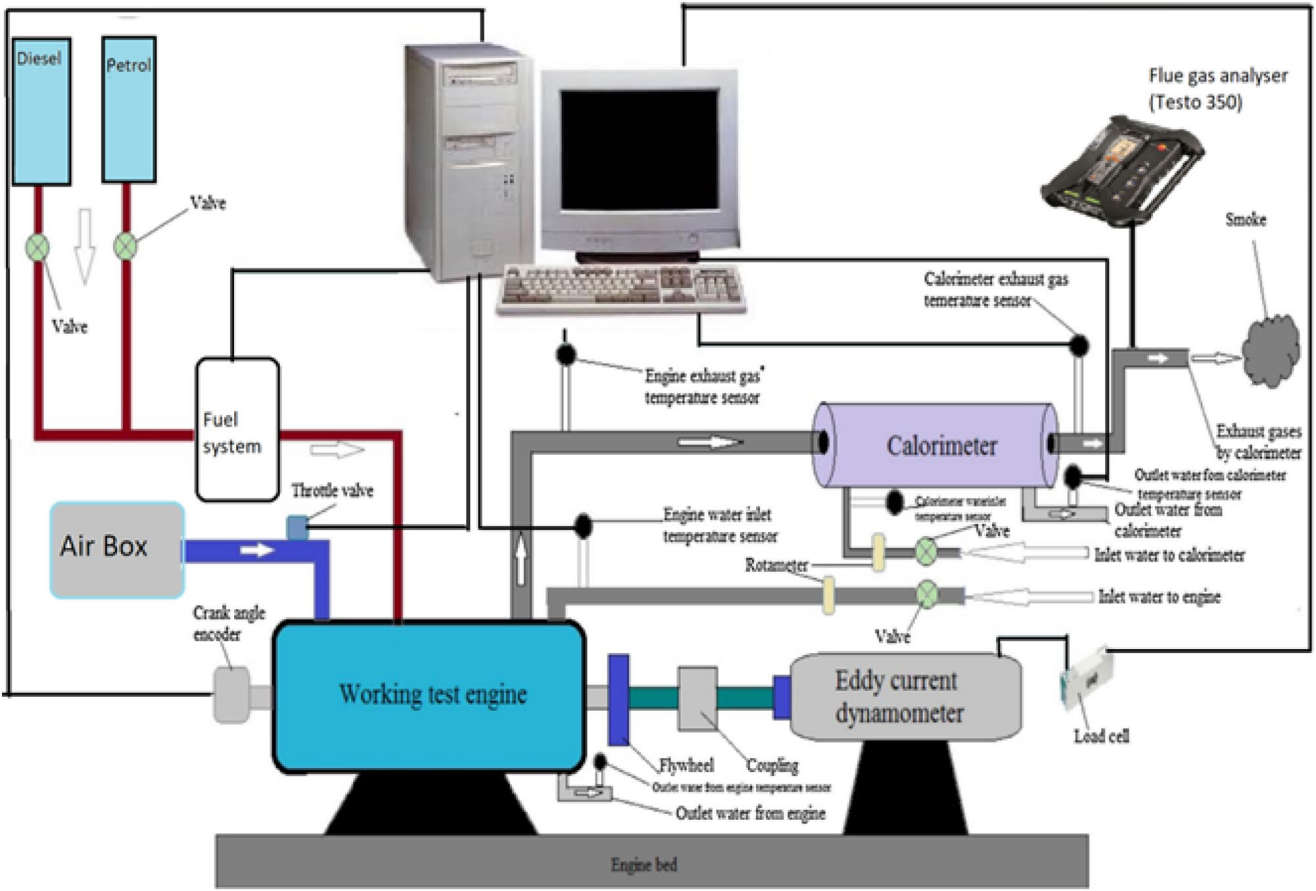
Experiemtal setup.

**Figure 2:**
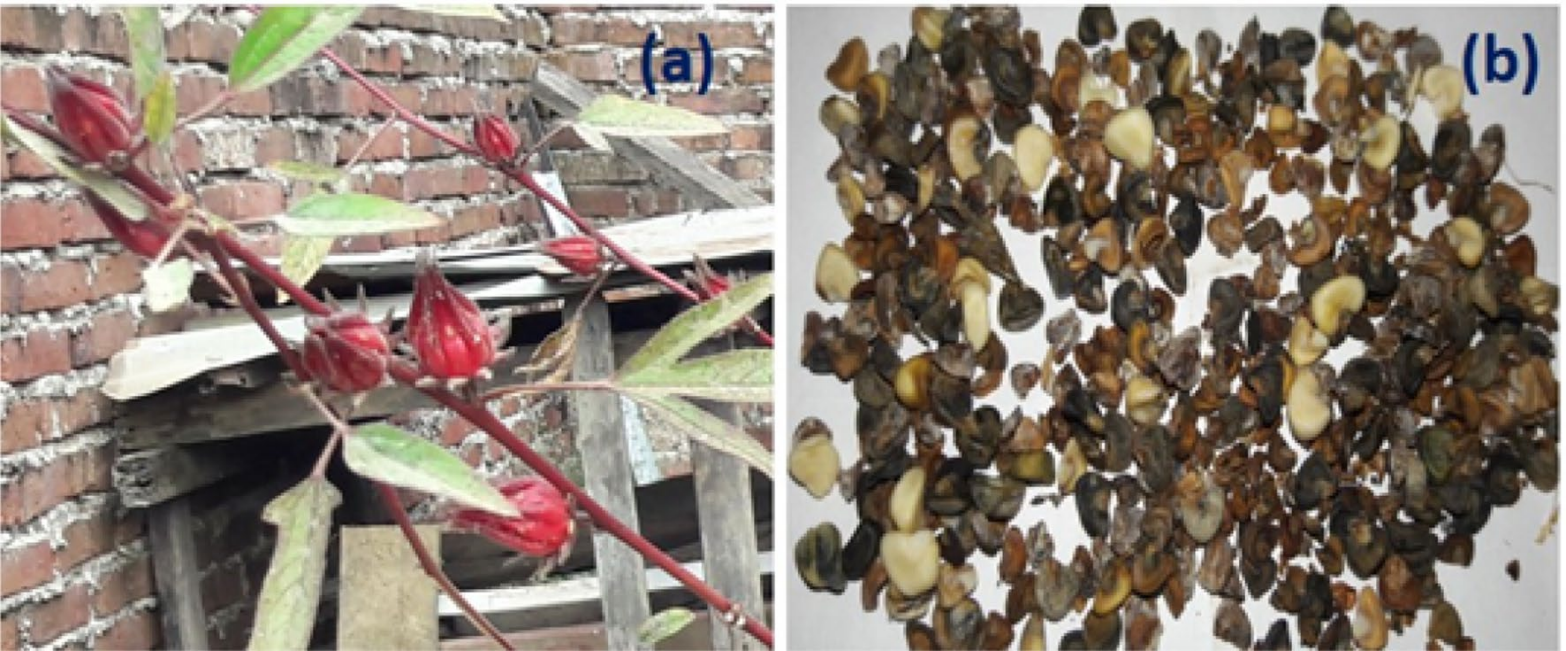
Roselle plant and seeds.

All experimental testing were carried out at the Department of Mechanical Engineering's Internal Combustion Engine Laboratory on a single-cylinder 4-stroke CI engine with the technical characteristics listed in Table 4. The test were conducted for Roselle biodiesel and its blends of LA20, LA40, LA60, LA80, and LA100 with FITs of 19°, 21°, 23°, 25°, and 27° bTDC, engine loadings of 25%, 50%, 75% and 100% with a fixed CR of 17.5:1 and constant engine speed of 1500 rpm. Changing FIT is accomplished by modifying the fuel injection pump. In this operation, standard instruments such as a screwdriver and a socket wrench are used. A time metre is also used to measure the precision of the timing modification. Figure 1 shows a schematic diagram of the experimental test setup. Figure 2 depicts a roselle plant with its seeds. A dynamometer was used to apply load to the test engine. Initially, engine was started with diesel fuel and the engine is allowed to warm-up for about 15–20 min to attain steady state condition. Testo-350 gas analyzer was used for measuring the exhaust emissions. Carbon dioxide (CO_2_), oxides of nitrogen (NOx), and smoke emission were measured from the exhaust gas by using the analyzer probe. The test matrix and acronyms used in the present research work for diesel, Roselle, and its blend are shown in Table 5.

**Table 4:** The detailed specification of test engine.

Engine parameters	Specifications
Make	Legion Brothers, India
Engine type	Four stroke, CI engine
Cooling	Water-cooled engine
Bore × Stroke	80 mm × 110 mm
No of cylinders	1
Compression ratio	17.5
Dynamometer	Eddy current dynamometer
Rated power@ 1500 rpm (k W)	3.5
Dynamometer	Eddy current dynamometer
Start of injection timing	19 to 27 °C bTDC
Connecting rod length	235 mm
Exhaust gas analyzer	Testo-350 FGA

**Table 5:** Test matrix and acronyms used for diesel and Roselle fuel operation.

Mode	Fuel used	Constant parameter		IT, °CA bTDC	Load	Acronyms used
		CR	Speed (rpm)			
Diesel	Diesel	17.5	1500	19	25%, 50%, 75%, 100%	B0
				21		
				23		
				25		
				27		
Biodiesel + Diesel	20% Roselle + 80% Diesel	17.5	1500	19	25%, 50%, 75%, 100%	LA20
				21		
				23		
				25		
				27		
Biodiesel + Diesel	40% Roselle + 60% Diesel	17.5	1500	19	25%, 50%, 75%, 100%	LA40
				21		
				23		
				25		
				27		
Biodiesel + Diesel	60% Roselle + 40% Diesel	17.5	1500	19	25%, 50%, 75%, 100%	LA60
				21		
				23		
				25		
				27		
Biodiesel + Diesel	80% Roselle + 20% Diesel	17.5	1500	19	25%, 50%, 75%, 100%	LA80
				21		
				23		
				25		
				27		
Biodiesel	100% Roselle	17.5	1500	19	25%, 50%, 75%, 100%	LA100
				21		
				23		
				25		
				27		

### Uncertainty analysis

All trial estimation, in general, is prone to errors and uncertainties. The choice of sensor, operating circumstances, calibration of the setup, test process, and observation could all contribute to the investigation's results being questionable. Table 6 summarises the equipment utilized in the experiment, including the estimation range and precision of the instruments. To reduce the error and verify the experimental accuracy, an uncertainty analysis for the experimental setup was required. To perform the uncertainty analysis, following method is discussed by^40–42,53^. The overall uncertainty analysis of the experimental was found out by using the following equation:

$$\begin{aligned} & {\text{Overall}}\,{\text{uncertainty}}\,\left( \% \right) \\ & \quad = {\text{square}}\,{\text{root}}\,{\text{of}}\,\left[ {\left( {0.2} \right)^{2} + \, \left( {1.0} \right)^{2} + \, \left( {0.15} \right)^{2} + \, \left( {0.5} \right)^{2} + \, \left( {0.2} \right)^{2} } \right. \\ & \quad \quad \left. { + \, \left( {1.0} \right)^{2} + \, \left( {0.15} \right)^{2} + \, \left( {1.0} \right)^{2} + \, \left( {1.0} \right)^{2} + \, \left( {1.0} \right)^{2} + \, \left( {0.5} \right)^{2} } \right] \\ \end{aligned}$$

**Table 6:** Uncertainty analysis of test engine.

S. No	Instrument	Percentage uncertainty
1	Load indicator	± 0.2
2	Speed sensor	± 1.0
3	Temperature sensor	± 0.15
4	Pressure sensor	± 0.5
5	Crank angle encoder	± 0.2
6	Smoke meter	± 1.0
7	Eddy current dynamometer	± 0.15
8	Fuel burette	± 1.0
9	Manometer	± 1.0
10	Test 350 gas analyser	
	CO_2_	± 1.0
	NO_X_	± 0.5

Total percentage of uncertainty =  ± 2.3%.

### ANN model

Empirical approximations of ICE operation are also essential for ICE research, as described in the introduction. Because experimental methods have limits in characterizing the whole operating range, we used ANN to construct an empirical model for application in prediction or optimization problems for the ICE operation using Roselle biodiesel.

**Figure 3:**
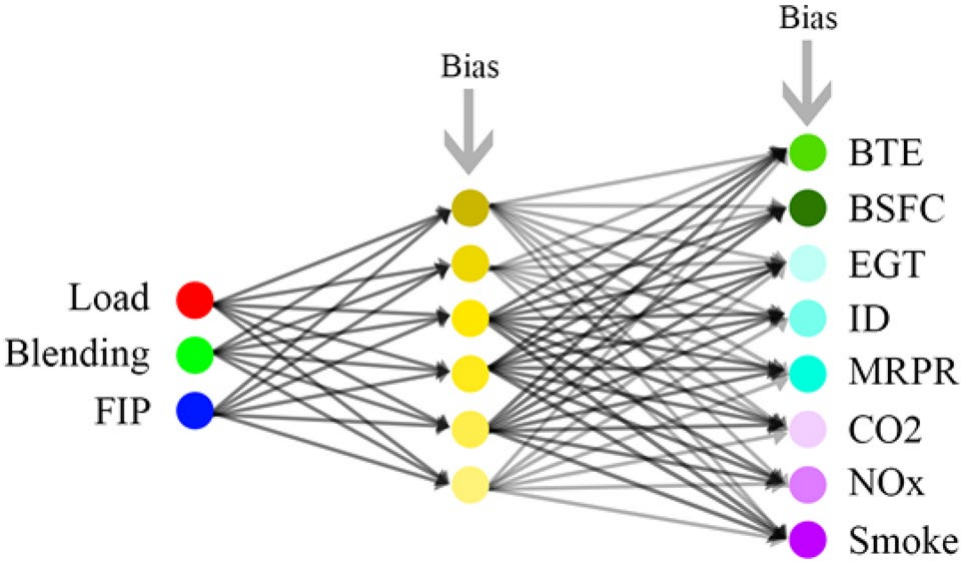
Schematic of ANN.

As summarised in Fig. 3 and Table 7, the employed ANN architecture had 3 input nodes representing loading, blending, and FIT. It had 10 output nodes to predict the associated performance variables of BTE, BSFC, and EGT, combustion variables of MRPR and ID, and emission variables of CO_2_, NOx, and smoke. The response of cylinder pressure and heat release rate was not included in the empirical analysis because of the different data structures. These two variables were operating at 100% loading condition only. The architecture had only one hidden layer which maps non-linear relationships from the input to the targets. And based on some thumb rules available in the literature^43,44^, the network was trained with 6 nodes in the hidden layer. Furthermore, having found satisfactory prediction results with the chosen architecture, no other architecture was checked for better performance and subsequent analysis was preceded with the 3-6-10 architecture. This also connoted to the suggestion of an empirically reduced model as will be discussed later. In addition, Levenberg–Marquardt backpropagation algorithm was employed as it is popularly reported to converge faster with minimum mean square error^45^. This was achieved with the function ‘trainlm’ from the Neural Network Toolbox available in MATLAB 2016a.

**Table 7:** ANN Settings used for modelling.

Parameter	Value	MATLAB function/syntax
Hidden layer neurons (h)	6	–
Topology	3-6-10	*fitnet(6)*
Data division (in %)	70%-15%-15%	*dividerand*
Data division (in data points)	84-18-18	–
Network type	feed-forward back-propagation	*feedforwardnet(hiddenSizes,trainFcn)*
Transfer function	Hyperbolic tangent sigmoid in hidden layer; linear in output layer	*tansig* in hidden layer; *purelin* in output layer
Training function	Levenberg Marquardt algorithm	*net.trainFcn* = *’trainlm’;*
Performance function	Mean square error	MSE
Learning function	Gradient descent with momentum weight and bias learning function	*learngdm*

The experiments with different operating conditions of 4 loading conditions, 6 blending percentages, and 5 FITs yielded 120 (4*5*6) operating conditions. Out of these, 70% corresponding to 84 conditions were randomly selected to train the network during which iterative adjustments were made to the weights and biases of the network. Validation of the network generalization was achieved with another 15% corresponding to 18 conditions. This same data subset was used for terminating the training when the network generalization didn’t improve. At the end of training, the remaining 15% corresponding to 18 conditions which were not involved at all in the training were used to evaluate the performance of network. These random divisions of data into these three sets were conducted using the ‘dividerand’ function available in MATLAB 2016a. In addition, prior to the training, the dataset was normalised as shown in Eq. (1):

1 $$Xn = \left( {Xi - Xmin} \right)/\left( {Xmax - Xmin} \right)$$

where Xn is normalised value of Xi; Xmin and Xmax are respectively minimum and maximum of the corresponding variable.

But a range of (0.05–0.95) was used instead of (0,1) to avoid possible arithmetic operations such as dividing by zero to it as it will be returned as ‘not a number’ (NaN) by the computational environment. In addition, it could help achieve faster training as transformations of extremely small values are avoided^46^. And with this, the network can avoid computing activation functions of extreme values without compromising the empirical accuracy. This was achieved with the modification of Eq. (1) into as Eq. (2):

2 $$Xn = \left( {b - a} \right).\left( {Xi - Xmin} \right)/\left( {Xmax - Xmin} \right) + a$$

where a and b are limits of the normalised value correspondingly substituted by 0.05 and 0.95.

And rearranging Eq. (2) to get the original value:

3 $${\text{Xi}} = {\text{Xmin}} + \left( {{\text{Xmax}} - {\text{Xmin}}} \right).\left( {{\text{Xn}} - {\text{a}}} \right)/\left( {{\text{b}} - {\text{a}}} \right)$$

For the presentation of results, the empirical model thus achieved was used to calculate the expected engine output under different operating conditions within the range of experiment. Each range of input variable was subdivided into additional 20 points to simulate the full range engine behavior. These replicated data points were used to analyze the empirical perspective of the study.

## Results and discussion

### Brake thermal efficiency

**Figure 4:**
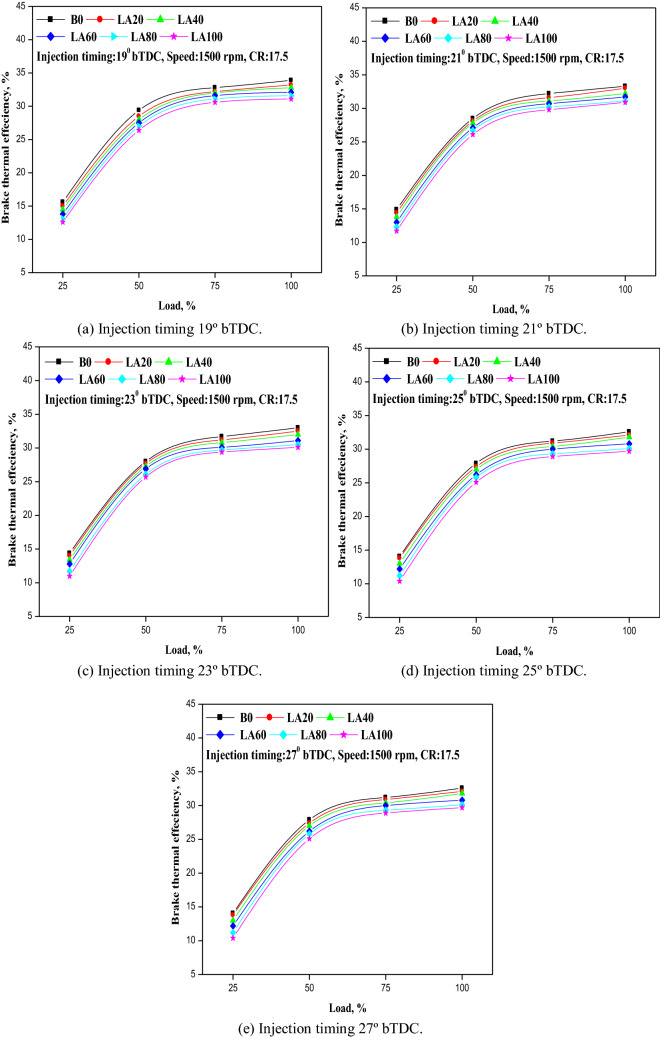
(**a–e**) The variation in BTE with different injection timing and engine load for test fuel blends.

The efficiency with which the engine transformed the chemical energy of the combustible fuel into usable work is defined. The fluctuation in BTE for the fuel blend samples with varying injection timing and engine load is shown in Fig. 4a–e. According to the results of the experiment, BTE dropped as FIT increased from 19° to 27° b TDC. In comparison to other biodiesel blends, BTE was higher for LA20 blend with retarding in FIT and decreased with advancement in FIT under higher load conditions, as reported by panneerselvam et al.^14^. Reducing the FIT may result in an early start of fuel combustion that lasts until the end of the power stroke. BTE was found to be greater with a 19° b TDC retarded FIT, with 15.65 percent at low load rising to 33.95 percent for diesel fuel at higher load, and 15.1 percent at low load rising to 33 percent for LA20 at higher load. While it decreased with improved FIT of 27° bTDC, 13.7 percent at lower load to 32.2 percent for diesel fuel at higher load, and 13.2 percent at lower load to 31.8 percent at higher load condition for LA20. LA20 and LA40 had BTEs that were approximately identical to diesel fuel, however, LA60, LA80, and LA100 had BTEs that were significantly lower.

### Brake specific fuel consumption

**Figure 5:**
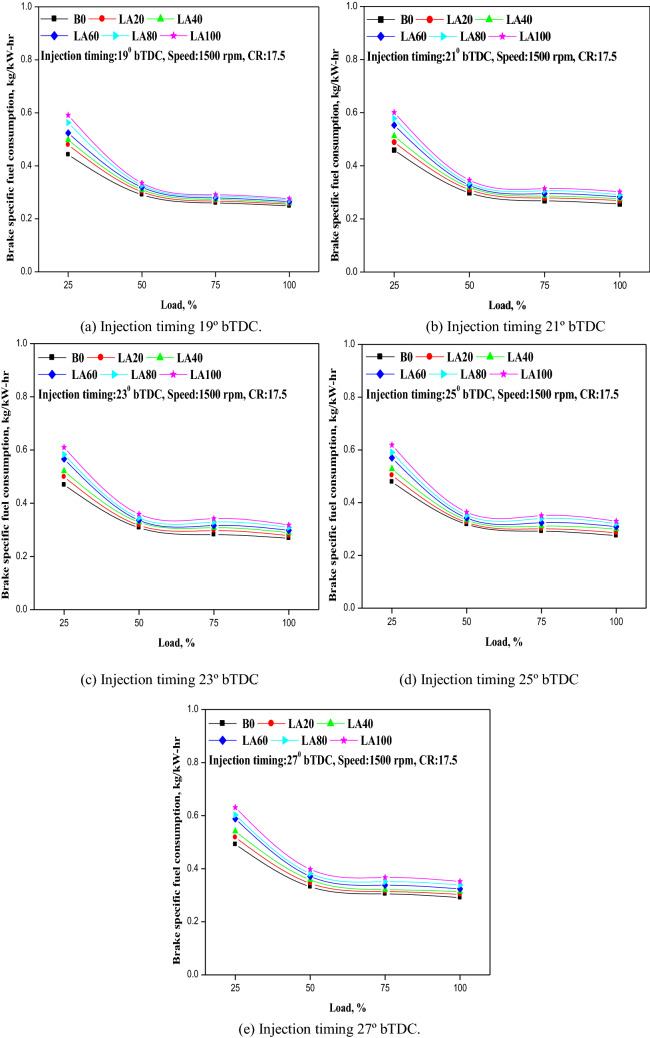
(**a–e**) The variation in BSFC with different injection timing and engine load for blend samples.

Brake specific fuel consumption is a measure of the engine's charge efficiency, and it's an important statistic to consider when determining how efficiently an engine's fuel is transformed into work. The fluctuation in BSFC for different tested fuels with different FIT and load is seen in Fig. 5a–e. The BSFC increased when the FIT increased from 19° to 27° b TDC. At standard FIT (23° b TDC), the BSFC for diesel fuel was 0.268 kg/kWh and 0.318 kg/kWh for LA100 at greater loads. The Roselle biodiesel as a fuel, for pure Roselle biodiesel as fuel (LA100) the BSFC increased to 0.331 kg/kWh and 0.352 kg/kWh with advanced FIT of 25° and 27° bTDC, and it decreased to 0.302 kg/kWh and 0.276 kg/kWh with retarded FIT with 21° and 19° bTDC compared to standard FIT. One possible reason for this increment in BSFC with advanced FIT could be due to longer ignition delay duration, and therefore more fuel getting accumulated in the process with increase in fuel consumption rate. Also, with increase in advancement of FIT from 19° to 27° bTDC, additional time was accessible for the combustion, and therefore that could prompt to better ignition of fuel. It was found that diesel fuel indicated lower BSFC for all FIT compared with Roselle and its blends. This could be because of the lower heating value and higher density of Roselle biodiesel and its mixes, which require more fuel to get the same power output as diesel. For all tested fuels, BSFC was found to be lowest with a retarded FIT of 19° b TDC and highest with an advanced FIT of 27° b TDC.

### Exhaust gas temperature

**Figure 6:**
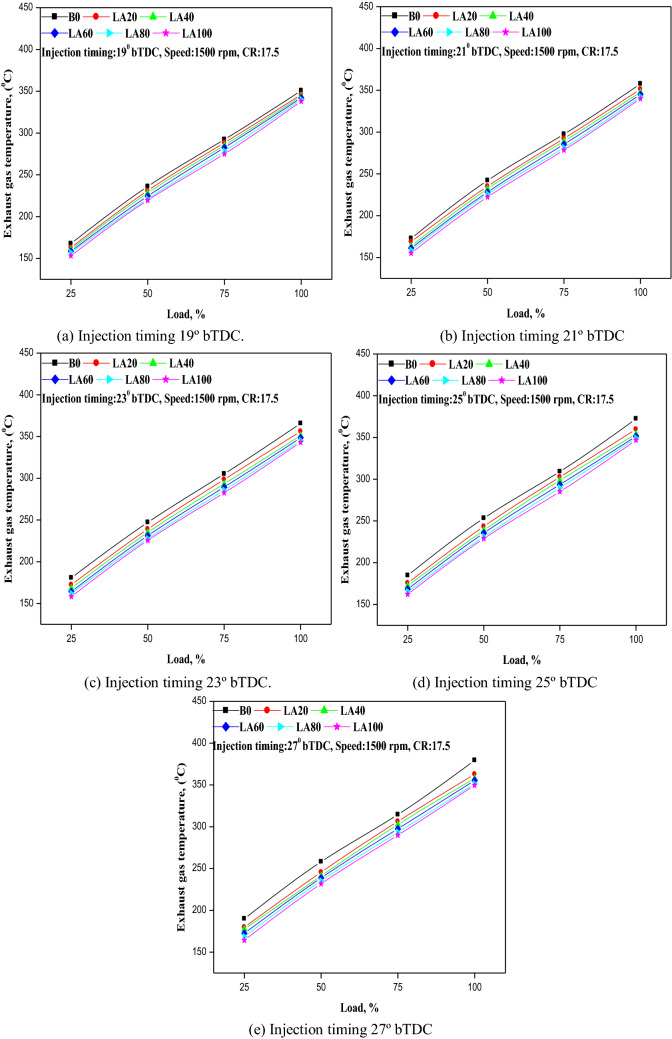
(**a–e**) The variation in EGT with different injection timing and engine load for different blends.

The temperature of the exhaust gas indicates the quality of combustion. The fluctuation of EGT with load at varied FIT is shown in Fig. 6a–e. With the progression of FIT from 19° to 27° b TDC, EGT rose. This could be because of advanced FIT, which causes a longer ignition delay period, resulting in higher cylinder temperature and pressure. EGT was measured at 350.8 °C and 338.1 °C for diesel and LA100 at 19° bTDC at full load. 357.8 °C, 365.8 °C, 372.4 °C, and 379.5 °C for diesel and 340.1 °C, 343.2 °C, 346.8 °C, and 349.7 °C for LA100 at 21° b TDC, 23° bTDC, 25° b TDC, and 27° b TDC, respectively. As a result, the testing results showed that FIT retardation lowered EGT by 3–6 °C, whereas accelerated FIT enhanced EGT for all tested fuels.

### Combustion characteristics

#### Cylinder pressure

**Figure 7:**
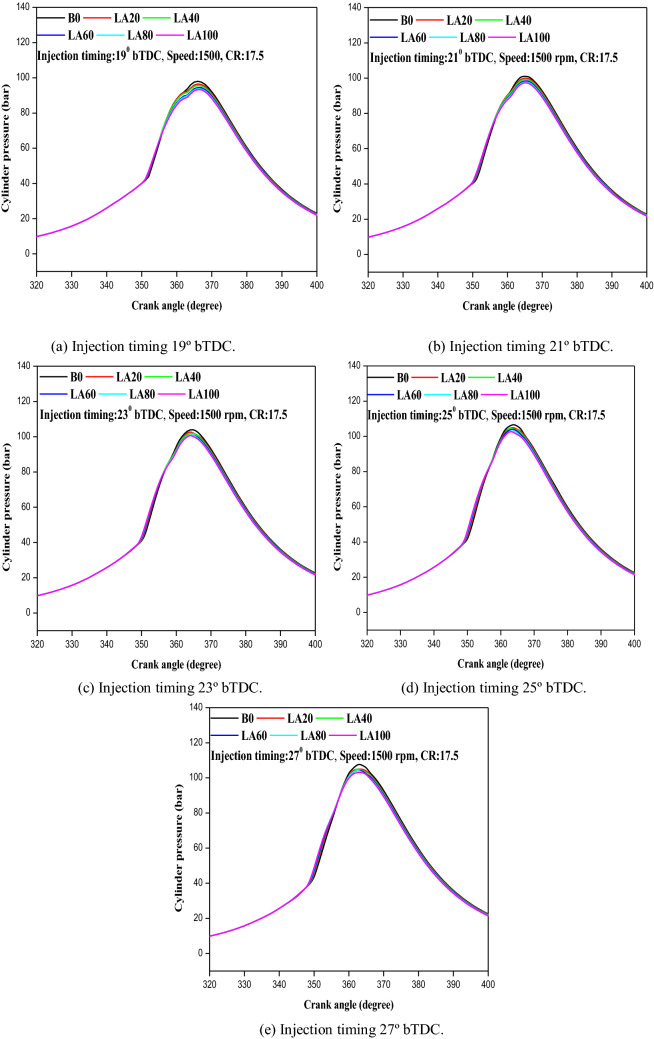
(**a–e**) The variation in-cylinder pressure with different FITs and engine load for considered fuel blends.

The cylinder pressure is necessary to study the behavior of combustion in the combustion chamber and also for examination of engine performance. The variation in cylinder pressure with crank angle at full load at different FIT is shown in Fig. 7a–e. It was found that cylinder pressure increased with increase in FIT. Diesel fuel indicated higher cylinder pressure for all engines operating conditions at different FIT compared to other biodiesel and its blends. At 19° bTDC, the cylinder pressure was recorded to as 98, 96.5, 95.7, 94.5, 93.9, and 93.3 bar for diesel, LA20, LA40, LA60, LA80, and LA100. Similarly, at 21°, 23°, 25°, and 27° bTDC cylinder pressure were measured as 101, 104, 106.8, and 108 bar for diesel and 97.1, 101, 102.2, and 103.2 bar for pure LA100. When comparing biodiesel and its mixes to diesel fuel, this clearly showed that cylinder peak pressure was lower for biodiesel and its blends. This could be owing to a shorter ignition delay time. With retarded FIT, the cylinder peak pressure dropped to 19° bTDC, but climbed with advanced FIT. For diesel fuel, the greatest cylinder peak pressure measured at 19° bTDC was 5 bars lower than that recorded at 23° bTDC. For LA20, LA40, LA60, LA80, and LA100, a 5–7 bar difference was noted. It also increased by about 4 bars with FIT for diesel, 3–4 bars for LA20, LA40, and LA60, LA80, and LA100. As a result, the enhanced FIT improved air–fuel mixing, resulting in a more efficient fuel combustion process. As a result of the prolonged ignition delay, the cylinder pressure increased.

#### Heat release rate

**Figure 8:**
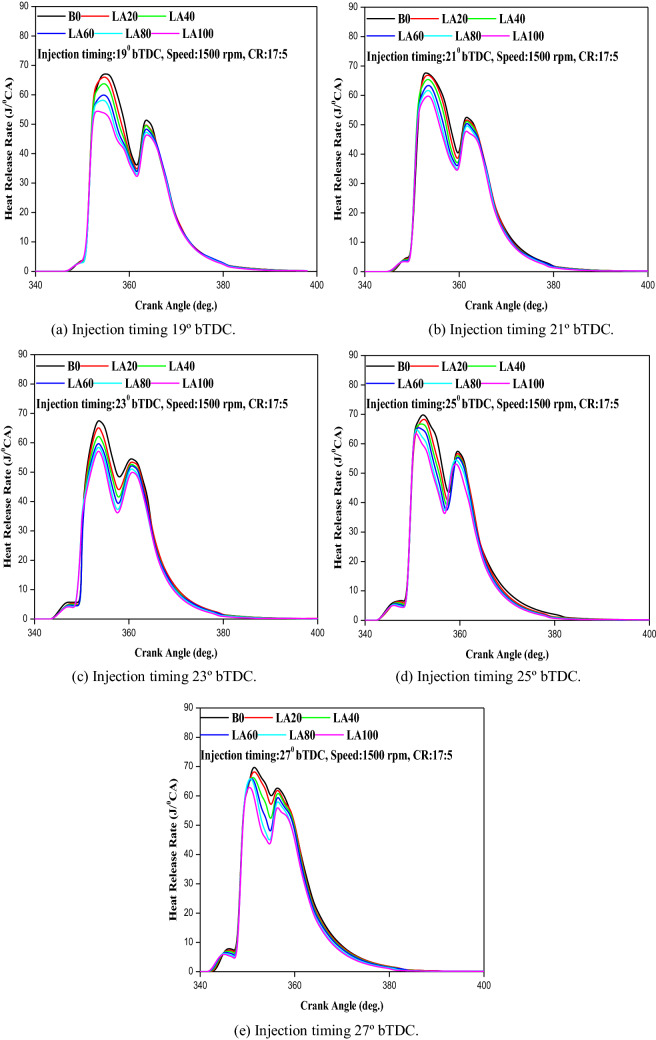
(**a–e**) The variation in HRR with different injection timing and engine load for blend samples.

At a certain crank angle, it is defined as the quantity of heat generated by the combustion mixture (instantaneous heat release rate). Figure 8 shows the relationship between HRR and crank angle at full load for various FITs (a–e). The HRR for all testing fuels was reduced when the FIT was retarded, according to the experimental results. The peak HRR for LA20 and LA100 was 66.3 and 54.8 J/°CA at 19° bTDC, 67.2 and 59.8 J/°CA at 21° bTDC, 66.4 and 58.2 J/°CA at 23° bTDC, 68.5 and 58.6 J/°CA at 25° bTDC and 68.9 and 63.1 J/°CA at 27° bTDC respectively. This could be due to decrease in biodiesel content in the fuel mixture resulting in increase in premixed combustion HRR for all FIT. This may be due to the higher viscosity and density of biodiesel fuel which also lead to poor mixing and atomization of fuel. At 19° bTDC, the peak HRR of diesel fuel was 67.2 J/°CA, whereas for 21° bTDC, 23° bTDC, 25° bTDC and 27° bTDC it was 67.9, 68.5, 70.2 and 73.2 J/°CA respectively. As a result, it is possible to deduce that FIT retardation reduced the ignition delay period, resulting in lower peak HRR and premixed combustion.

#### Ignition delay

**Figure 9:**
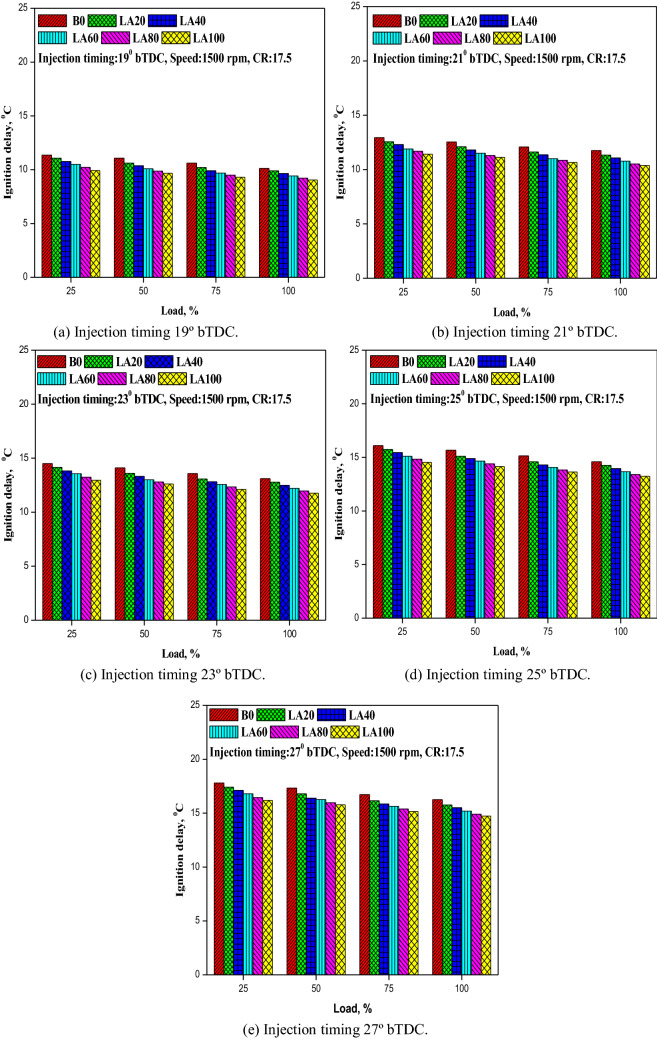
(**a–e**) The variation in ID with different FITs and engine load for considered fuel blends.

The ignition delay is the time gap measured in degrees crank angle between the start of fuel injection and the start of combustion. Figure 9 depicts how ignition latency changes with various engine loads and FITs (a–e). As indicated in the graph, the ignition delay decreased as engine load grew and FIT advanced. Longer ignition delays resulted from increased advanced FIT, resulting in more fuel being collected inside the engine cylinder and more premixed combustion^50,51^. Temperatures and pressures increased as a result. This explains why the ignition delay period for diesel fuel was longer than for Roselle biodiesel and its mixes at all operational FITs. The ignition delay for LA100 was reduced to 9.05°, 10.38°, 11.76°, 13.23°, and 14.73° at 19°, 21°, 23°, 25°, and 27° bTDC, compared to 10.12°, 11.75°, 13.1°, 14.6°, and 16.25° for diesel fuel at 19°, 21°, 23°, 25°, and 27° bTDC.

#### Maximum rate of pressure rise

**Figure 10:**
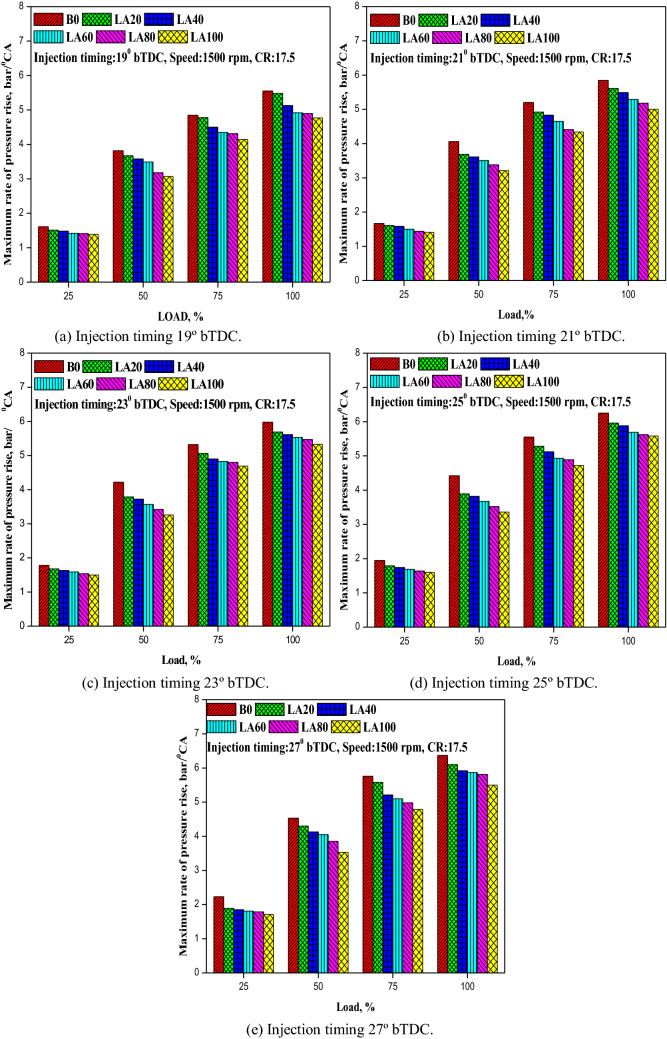
(**a–e**) The variation in MRPR with different FITs and engine load for considered fuel blends.

Figure 10 depicts the fluctuation in MRPR with load at various FITs (a–e). For all of the fuels tested, the MRPR increased as the load and FIT increased. Diesel fuel showed a larger pressure rise than the other tested fuels in all engine running situations. This increase in pressure could be attributed to advanced FIT injecting more fuel, which takes longer to charge the fuel–air combination and creates a maximum pressure when the piston reaches TDC. The MRPR for LA100 at full load was 4.7, 5.3, 5.8, 5.62, and 5.7 bar/°CA, respectively, which is lower than diesel fuel by around 5.55, 5.85, 5.98, 6.25, and 6.37 bar/°CA at 19°, 21°, 23°, 25°, and 27° b TDC.

### Emission characteristics

#### Smoke emission

**Figure 11:**
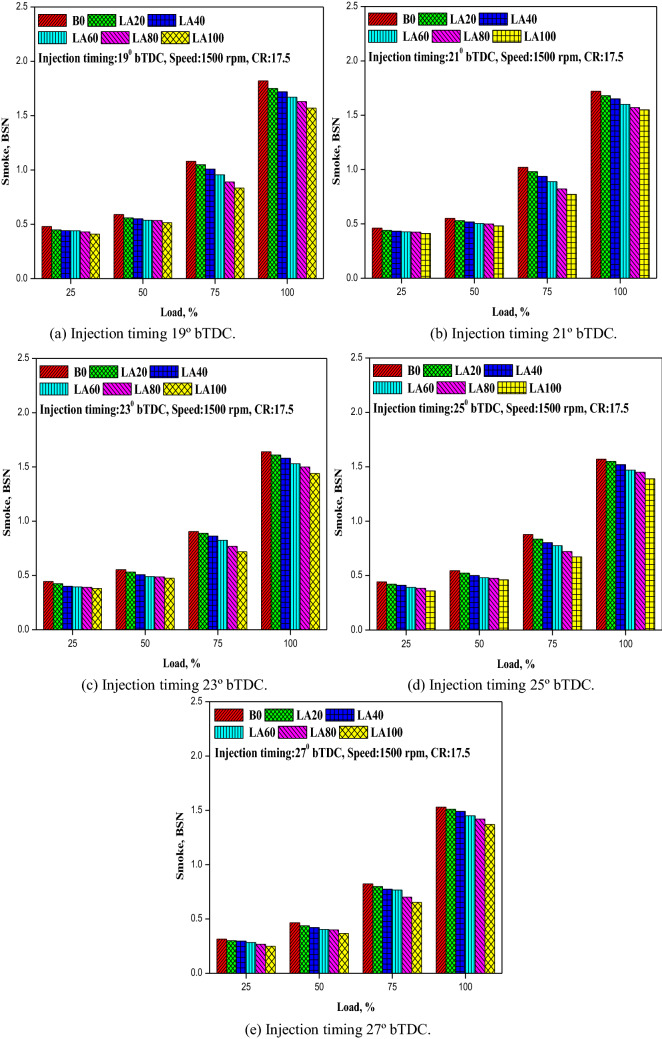
(**a–e**) The variation in smoke emision with different FITs and engine load for considered fuel blends.

Figure 11 depicts the variance in smoke emission as a function of engine load and FIT (a-e). The amount of smoke produced grew as the engine load increased, but decreased with advanced FIT. At all FITs, diesel fuel produced the most smoke, followed by Roselle biodiesel and its blends. For example, as presented in figure increased FIT, the smoke emission decreased by 12.90%, 13.81%, 14.23%, 14.78%, and 15.03% for LA20, LA40, LA60, LA80, and LA100 at full load condition respectively. It could be due to the presence of more oxygen content in the biodiesel, therefore the fuel rich zone decreased and also restricted the formation of smoke emission^47,48^. Smoke was produced as a result of incomplete charge combustion in a fuel-rich zone. At 19°, 21°, 23°, 25°, and 27° bTDC at full load, smoke emission was determined to be 1.82, 1.72, 1.64, 1.57, and 1.53 BSN for diesel fuel compared to 1.57, 1.55, 1.44, 1.39, and 1.37 BSN for LA100.

#### CO_2_ emission

**Figure 12:**
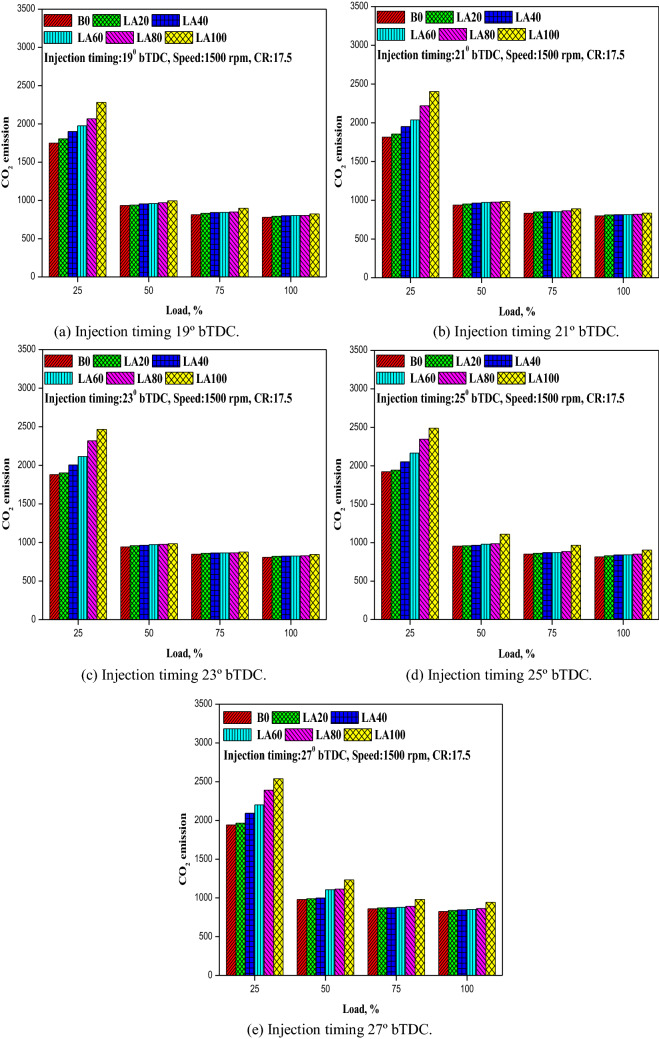
(**a–e**) The variation in CO_2_ emision with different FITs and engine load for considered fuel blends.

Figure 12 depicts the variance of CO2 emissions with load at various FITs (a-e). For all tested fuels, CO2 emissions increased as FIT increased and reduced as engine load increased. The CO2 emissions from diesel gasoline with a delayed FIT were lower. CO_2_ emissions for LA100 were 823.2, 833.84, 845.6, 905.1, and 943.2 g/kWh at 19°, 21°, 23°, 25°, and 27° bTDC, respectively, compared to diesel fuel emissions of 779.4, 798.2, 807.23, 816.2, and 824.23 g/kWh at 19°, 21°, 23°, 25°, and 27° bTDC. This could be owing to a longer ignition delay, which allows for better mixing of the air–fuel mixture. This could create a fuel-rich zone, resulting in improved fuel combustion and increased CO2 emissions.

#### NOx emission

**Figure 13:**
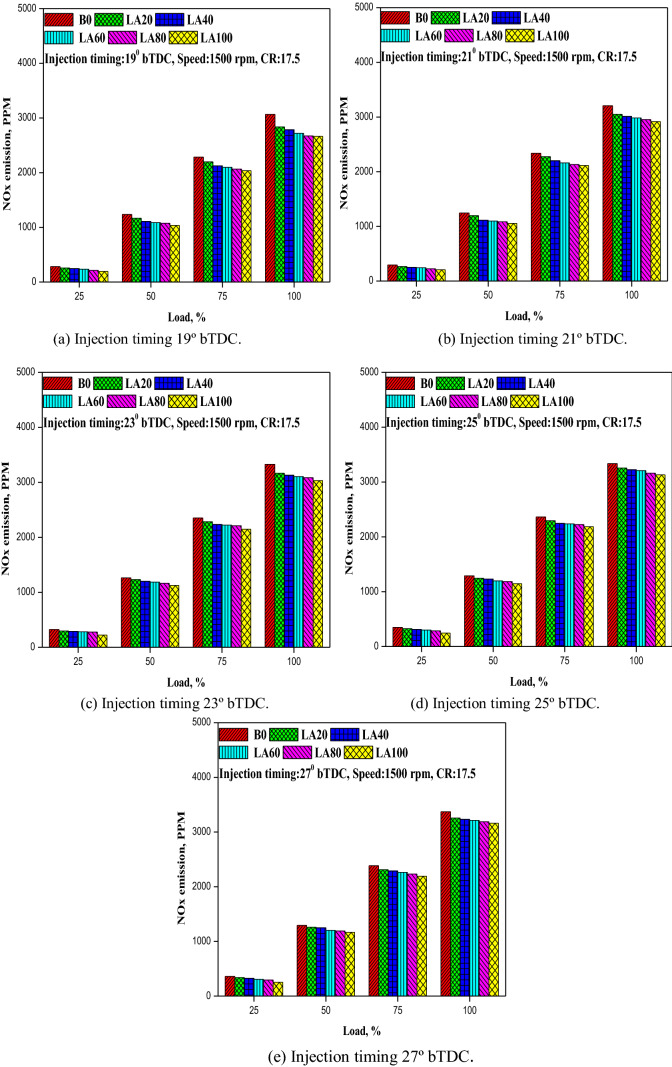
(**a–e**) The variation in NO_X_ emission with different FITs and engine load for considered fuel blends.

As seen in Fig. 13, NOx emission varies with load at various FITs (a–e). Increased FIT from 19° to 27° bTDC resulted in increased NOx emission for all tested fuels, as seen in the figure. For all FIT, diesel fuel released more NOx than biodiesel and its blends, according to Singh et al.^48^. This could be explained by the fact that as FIT increases, the ignition delay increases, lowering the gas temperature at early fuel injection. Because of the prolonged ignition delay, the amount of fuel burned in the premixed combustion phase increases, resulting in higher cylinder gas pressure and temperature, as well as increased NOx emissions^49^. At 19° bTDC, higher NOx emission of 255.58 ppm was observed for LA20 at low load, and 2835.8 ppm at full load. Similarly, at 21° bTDC, 23° bTDC, 25° bTDC, and 27° bTDC, it was 267.4, 299.36, 326.7, and 338.2 ppm at low load whereas 3050.1, 3165.5, 3254.2 and 3257.7 ppm at full load condition respectively. Due to the lower calorific value and higher viscosity of diesel fuel, all other biodiesel blends produced lower NOx emissions than LA20.

#### ANN model performance

**Figure 14:**
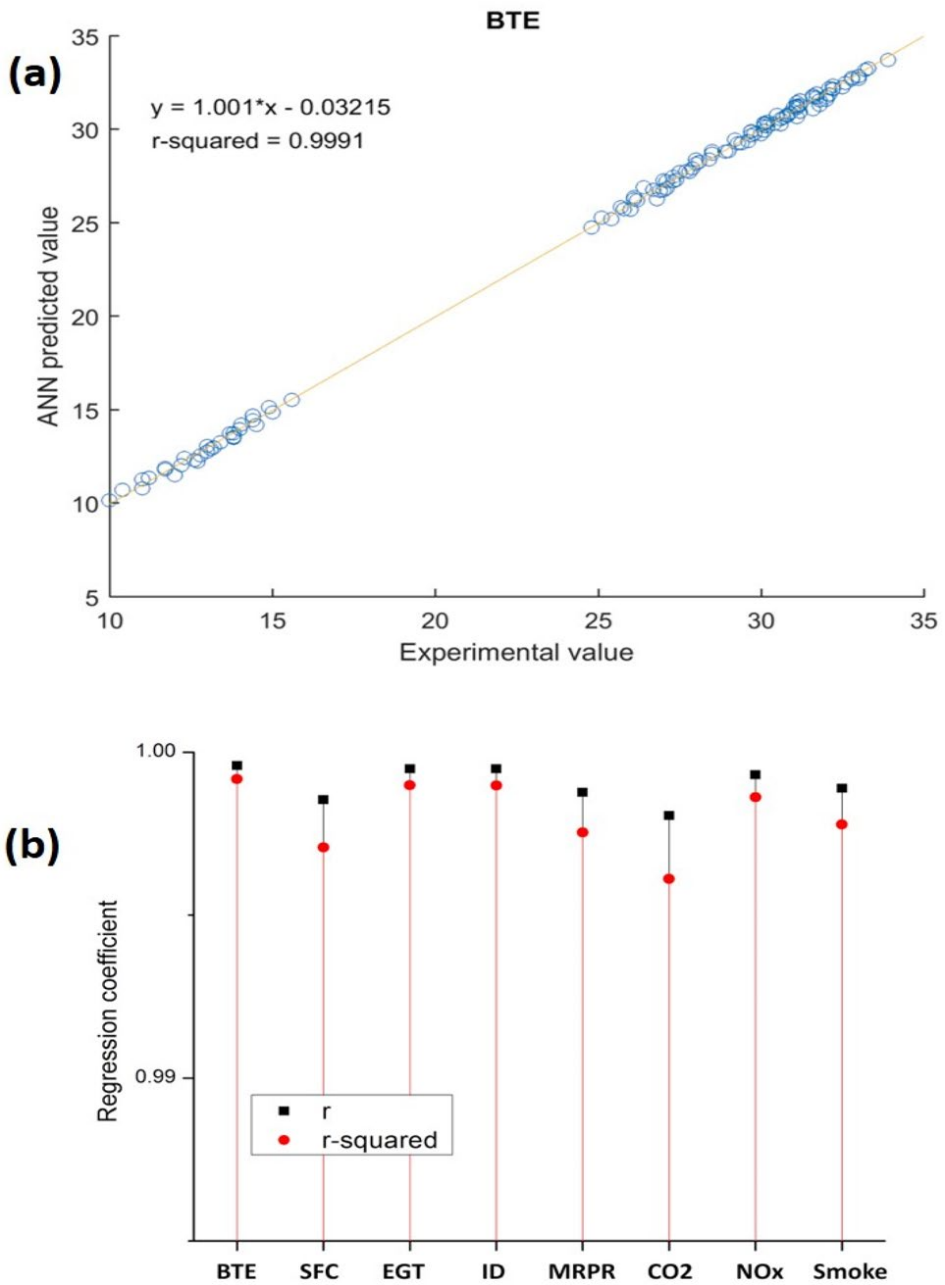
(**a,b**) The the prediction accuracy for all the responses.

As it was indicated by a significantly high Pearson correlation coefficient (value for representative variable of BTE as r = 0.9996), the ANN model could predict with satisfactory accuracy. It is visualized in Fig. 14a where the slope of the linear polynomial fitted to the scatter data points is very close to 1 (1.001). Here, a slope of unity would mean that the network prediction was exactly the same as the experimental value. Further, the prediction accuracy for all the responses is shown in Fig. 14b where the mean and standard deviation of r-value and r-squared-value are presented. For all the 10 responses, r-value and r-squared value were 0.9990 ± 0.0005 and 0.9980 ± 0.0011 (as mean ± standard deviation) respectively.

#### Computational implications

As it was evident from the engine behavior reported here, complex non-linear relationship existed among the several output responses with respect to the operating conditions. These complex relationships reinforce the need for alternative approaches for modelling ICE operation. The requirement becomes dire when pragmatic industry feasible solutions are required for developing complete response surfaces. This can be delivered by ANN as empirical compromise that also accounts for the multivariate interactions among the variables of interest at higher dimension. Such complete responses become mandatory for problems like optimisation where it is very important to achieved global solution instead of local solutions.

**Figure 15:**
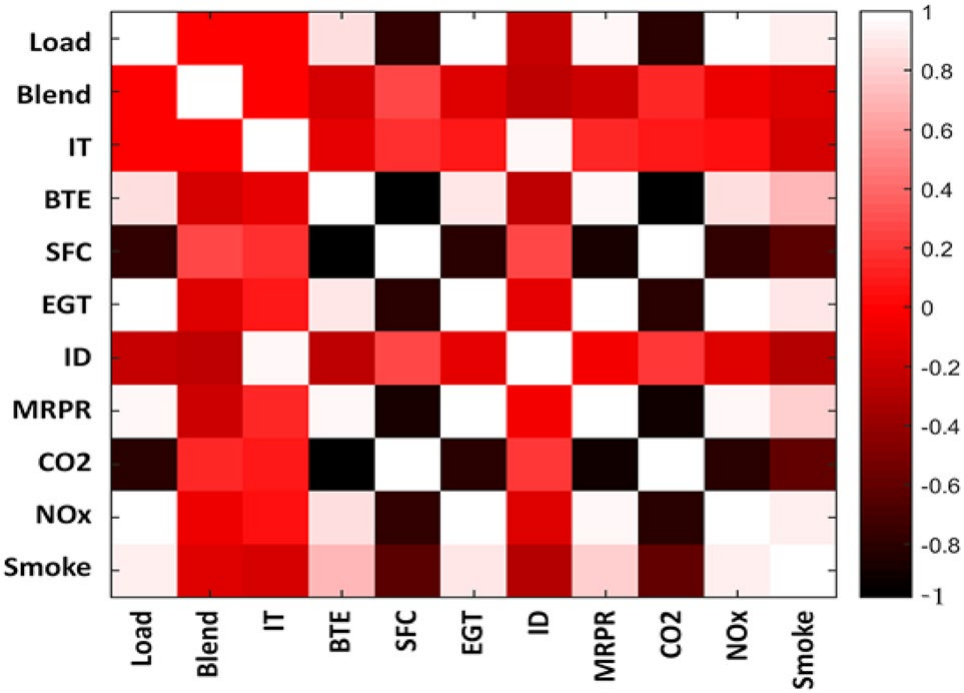
despite the non-linearity in the reported responses.

This empirical modelling further lead to plausible empirically reduced models of ICE operation. As shown in Fig. 15, despite the non-linearity in the reported responses, significant correlations were observed among the variables of study. As also suggested by^37^ collectively representing such correlated responses by a single variable would lessen the associated computational cost of prediction or modelling. For example, the representative variable EGT exhibited very high correlation with other variables of BTE, MRPR, and NOx. As seen in Fig. 15, such empirical redundancy can be harnessed by using simple linear or quadratic relations to represent those dependencies. Based on the degree of accuracy desired in the prediction of the responses, many variables with degrees of polynomial can be chosen to be substituted by only one representative response variable.

## Conclusion

The technical feasibility of using Roselle biodiesel as a substitutive fuel for CI engine had shown that:

- For all engine operating circumstances, BTE was higher for diesel fuel than biodiesel and its blends, and it increased with delayed FIT.
- At full load, the BSFC for LA100 was 17.3% higher at advanced FIT compared to 9.78% at retarded FIT. With improved FIT, Blend LA20 showed greater EGT, ignition delay, and maximum rate of pressure rise.
- When compared to diesel, NOx emissions from biodiesel and its blends were lower. In addition, with improved FIT, NOx emissions increased. With enhanced FIT, CO_2_ emissions increased while smoke emissions dropped.
- In addition, for further examination of empirically reduced models, an ANN model was created to predict engine characteristics. The ANN model was able to predict satisfactorily with an average r-value and r-squared value of 0.9990 ± 0.0005 and 0.9980 ± 0.0011 (as mean ± standard deviation) respectively for all 10 responses.
- Empirical redundancy in the dataset can be used by developing a substitutive variable to represent a set of strongly correlated variables using simple linear or quadratic relationships.
- Finally, this study characterized the performance, combustion, and emission of biodiesel generated from Roselle in engines. Because of the diverse engine responses to diesel fuel, Roselle can be used as a diesel fuel alternative in terms of technological feasibility. Roselle has a significant potential as an economically feasible alternative fuel for CI engines due to its growability and commercial viability.

### Ethical approval

The plant study was done in accordance with relevant guidelines and regulations. And also proper permissions/approval are taken for carrying out this study.

## Abbreviations

ANN: Artificial neural network
B0: Diesel fuel 100%
BTE: Brake thermal efficiency
BSEC: Brake specific energy consumption
BSFC: Brake specific fuel consumption
bTDC: Before top dead centre
CO_2_: Carbon dioxide
CI: Compression ignition
CA: Crank angle
CO: Carbon monoxide
CR: Compression ratio
EGT: Exhaust gas temperature
EGR: Exhaust gas recirculation
HC: Hydrocarbon
FIT: Fuel injection timing
FIP: Fuel injection pressure
ICE: Internal combustion engine
LA20: Roselle 20% + Diesel 80%
LA40: Roselle 40% + Diesel 60%
LA60: Roselle 60% + Diesel 40%
LA80: Roselle 80% + Diesel 20%
LA100: Roselle biodiesel 100%
MRPR: Maximum rate of pressure rise
NOx: Oxide of nitrogen
PM: Particulate matter
RPM: Rotation per minute
TDC: Top dead centre
UHC: Unburned hydrocarbon
